# Enhancing Industrial Automation in Industry 4.0: Integrating Node-RED with Real PLCs for Analog and Digital Signal Simulation.  

**DOI:** 10.12688/f1000research.176034.2

**Published:** 2026-07-17

**Authors:** Firas Ahmed Hussein, Mohammad Tariq Yaseen, Mohammed Obaid Mustafa

**Affiliations:** 1 Department of Electrical Engineering ,College of Engineering, University of Mosul, Mosul, Nineveh Governorate, 41001, Iraq; 2 Department of Communications and Intelligent Digital Systems Engineering, University of Mosul, Mosul, Nineveh Governorate, 41001, Iraq

**Keywords:** Node-RED Tool, Real PLC, Industrial Internet of Things (IIOT), Analog and Digital Signals Simulator.

## Abstract

**Background:**

Within the framework of Industry 4.0, industrial automation plays a pivotal role in the development of smart systems. Programmable logic controllers (PLCs) are the core components of these systems, enabling precise control of various industrial processes, while recent advancements emphasize the integration of open-source platforms to enhance system monitoring, simulation, and testing capabilities.

**Methods:**

In this study a real PLC from Allen-Bradley has been used, specifically the MicroLogix 1400, and also used Node-RED (Node-RED is an open-source, multitasking, easy-to-use graphical programming environment based on composing flows consisting of groups of specialized nodes to perform specific tasks) as a standalone simulator for analog and digital input and output signals. The presented study demonstrates how Node-RED can be used as an advanced PLC simulator to simulate analog and digital signals, either manually or automatically, enhancing the role of industrial automation in Industry 4.0.

**Results:**

The results demonstrated that Node-RED is a practical simulator for both analog and digital signals, with stable communication and consistent response behavior. and can be used to simulate complex industrial environments, enabling comprehensive testing and continuous improvement to achieve optimal results before implementing PLC systems in real-world settings.

**Conclusions:**

The research confirms that connecting the Node-RED tool with a real PLC is a useful, cost-effective solution and practical technique to simulate analog and digital (input/output) signals in industrial automation systems before implementing the PLC in the real world. This strategy improves cold commissioning efficiency and system reliability; these are the aims of Industry 4.0.

## Introduction

The Fourth Industrial Revolution, referred to as Industry 4.0 (I4.0), aims to integrate traditional systems such as the Programmable Logic Controller (PLC), which is characterized by ease of operation, high speed, excellent reliability, noise resistance, and outstanding stability, making it highly beneficial for automation control applications ^1, 2^ with modern technologies including the Industrial Internet of Things (IIoT). IIoT enables remote access to industrial physical objects anytime and anywhere with an internet connection ^3, 4^ and cloud computing, which combines various disciplines such as sensing technology, data processing, and analytics to enhance efficiency and productivity. ^5, 6^ Node- RED, an open-source development environment created by IBM, ^7^ is flow-based and facilitates integration between PLCs and databases. ^2^ Therefore, Node-RED, based on the Node.js engine using JavaScript, is considered one of the good solutions for system integration. ^8^ Node-RED has recently gained significant global popularity due to its simplicity and versatility. ^7^ Recently, some manufacturers of programmable logic controllers (PLCs) have promoted the use of virtualized PLCs (vPLCs) hosted within edge computing platforms. ^9^ However, traditional PLC simulators face significant challenges in effectively supporting analog signals, limiting their ability to simulate complex work environments. In this study, we propose a model and present Node-RED flows to use it as a simulator for analog and digital signals (input/output) and integrate it with real PLCs. ^9^ The integration is achieved via the EtherNet/IP protocol, creating a real simulation of the PLC as if it were installed in a real environment, where it sends and receives signals to and from various sensors at the worksite. The signal values can be manually modified through a user interface in Node-RED called the Dashboard. ^7^ Analog input and output signals linked to the PLC can also be automatically adjusted, mimicking real signals interacting with any analog sensor on-site using custom JavaScript codes. ^8^ Thus, the state of the sensors is evaluated, the PLC’s strength and stability are tested, and weaknesses in the programming software (RSLOGIX 500 in this study) are addressed to provide the necessary solutions before deploying the PLC in a real environment. The research successfully achieved its goal of integrating traditional control systems with advanced technologies of Industry 4.0. It also confirmed that IIoT offers many advantages for industry in terms of time, cost, and production efficiency. Most importantly, applying IIoT represented by the Node-RED platform in this research facilitates industrial processes. ^3^

This research aims to enhance the integration of analog and digital signals and evaluate their role in improving industrial performance within the framework of Industry 4.0. Additionally, the study seeks to understand how Node-RED can be used as a “translation” tool that enables legacy PLC systems to communicate over the internet with Industry 4.0 devices.

**Figure 1.**
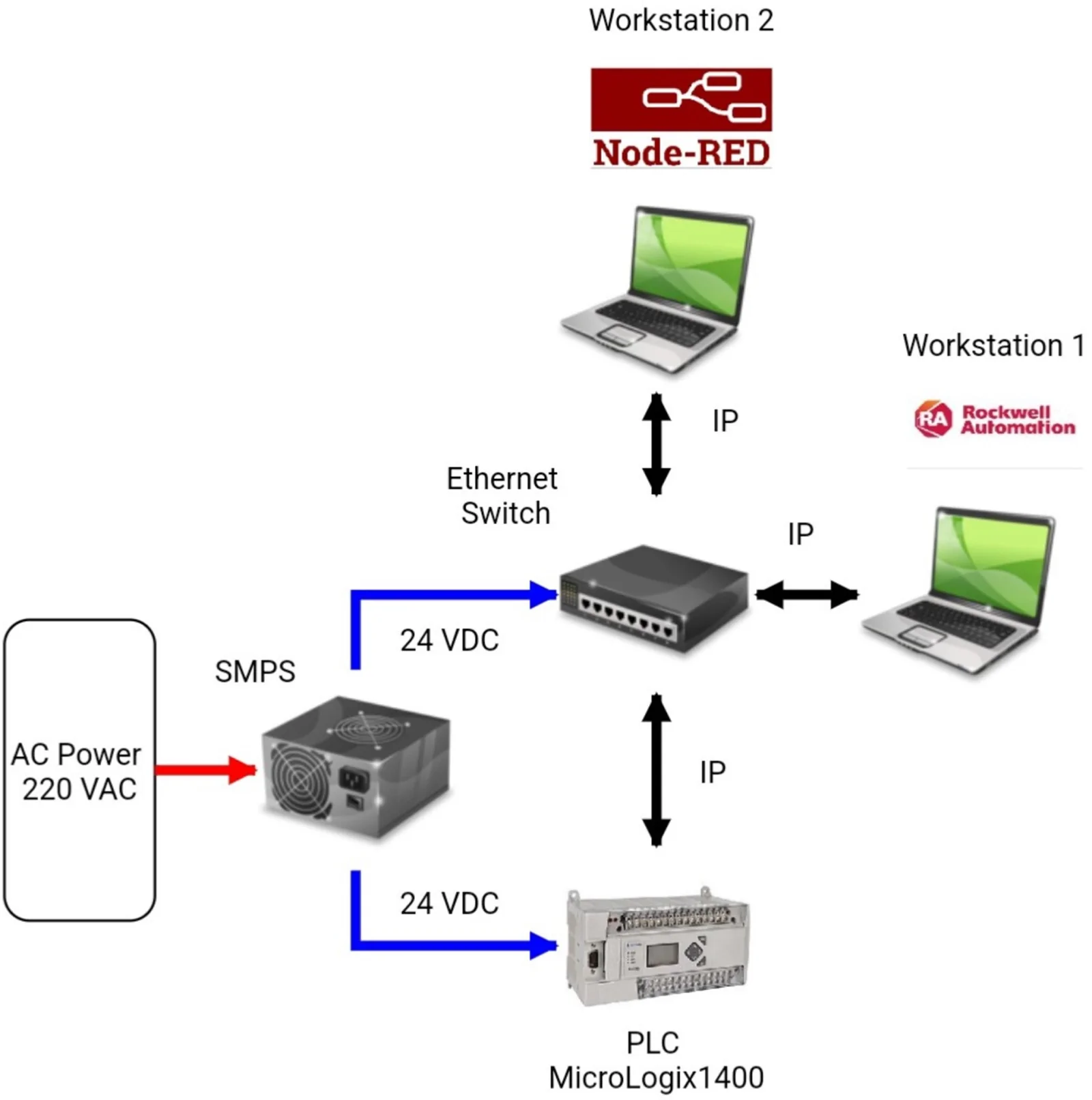
The Architecture of the System: This figure illustrates the connections between the system components in this study, the relationship between them, and how they integrate into the simulation and control process.

The significance of this research lies in highlighting the effectiveness of Node-RED as a reliable simulator for analog and digital signals (input/output) transmitted from sensors and field devices to programmable logic controllers (PLCs). The study specifically focuses on Allen-Bradley PLCs by presenting a practical model that demonstrates how to connect and operate Node-RED with a real PLC (AB MicroLogix 1400), as shown in Figure 1.

### Related work

Studies indicate that Node-RED is an effective solution for bridging traditional and modern control systems, offering a visual interface and advanced data analysis. Research shows that integrating Node-RED with PLCs helps overcome the limitations of traditional simulators, making it a preferred choice for control systems in Industry 4.0. A. Hijazi et al. in ^2^ built a complex connection between PLCs, Node-RED, and four different databases (MSSQL, MySQL, MongoDB, and Apache Cassandra). Faisal Bahri et al. in ^3^ successfully achieved remote control and monitoring of IoT-based induction motors using Node-RED, which performed as expected. Sri Sudha Vijay Keshav Kolla et al. in ^5^ created a simple framework to retrofit legacy machines using external sensors to collect data and transmit it to cloud databases for analysis and monitoring purposes. Gabriel Gaspar et al. in ^6^ focused on upgrading an old climate chamber to meet current requirements using a Node-RED-based solution. This solution entirely replaces the outdated system while providing options for low-cost hardware and software additions. Massimiliano Gaffurini et al. in ^9^ presented methodology, practical experiments, and a number of metrics to establish the communication and transfer data between PLCs and vPLCs. Daniel Ribeiro de Sousa et al. in ^10^ proposed a model and presented Node-RED flows to achieve Industry 4.0 capabilities on S7–300 Siemens PLCs connected via a PROFIBUS network. Antonio Morán et al. in ^11^ created an educational system that allows students to access remotely using virtual machines configured with all the software needed to learn industrial automation. Mohamed Saban et al. in ^12^ introduced a dedicated smart farming system utilizing low-cost, low-power, long-range wireless sensor networks based on IoT and LoRa technologies. G. Esteves Coelho et al. in ^13^ proposed a methodology to integrate modern systems into legacy infrastructure. Likewin Thomas et al. in ^14^ designed a low-cost home automation system that supports the Internet of Things, IoT. The Node-RED platform was used, which uses nodes to represent tasks and processes. This system can operate various household appliances, including sockets, from anywhere. Wireless Sensor Network (WSN) technology records data and uploads it to a web server from each room, while these technologies communicate using the MQTT protocol. Francisco Javier Folgado et al. in ^15^ presented a reference document and detailed steps for the design and deployment of Industry 4.0 based automation and supervision systems. Erik Kučera et al. in ^16^ prepared educational case studies of modeling and controlling a virtual system using PLC and linking it to a cloud-based control and monitoring application, in order to achieve the goal of digitizing production processes. M. M. Ahmadpanah et al. in ^17^ Proposed solutions to address security risks in Node-RED and protect against malicious and unwanted flows and nodes. Onwuegbuzie et al. in ^18^ presented an integrated platform combining Node-RED with IoT analytics for real-time data processing and visualization, demonstrating its effectiveness in industrial IoT applications. Nugraha et al. in ^19^ used the effectiveness of integrating the temperature sensors of an IoT into the Node-RED platform to monitor transformers in the laboratory, for predictive maintenance and energy optimization. Sousa et al. in ^20^ upgraded legacy control systems to Industry 4.0 standards using Node-RED and OPC-UA as a low-cost solution.

This research presents a new contribution on the role of the Node-RED tool in simulating analog and digital control signals, highlighting its significant potential that has not been exploited compared to previous studies, using it as an analog and digital I/O simulator when integrated with a Rockwell Automation PLC, specifically the Allen-Bradley MicroLogix 1400 PLC, by using the Ethernet/IP protocol.

### Comparison with previous research

In order to better place this study within the body of research already available, an organized comparison was made between it and the most current studies that were relevant, specifically the research by Sousa et al. on “Upgrading Legacy Systems for Industry 4.0 with Node-RED and OPC-UA.” The comparison is mostly about important technical factors like signal types, communication methods, how well they work in the real world, and data analysis, as shown in Table 1.

**Table 1.** Comparison between this study and previous work: This table shows the main differences and similarities, as well as the research gaps that this study seeks to resolve.

Sousa et al. in ^20^ (Upgrading Legacy Systems for Industry 4.0 with Node-RED and OPC-UA)	This Study (Enhancing Industrial Automation in Industry 4.0: Integrating Node-RED with Real PLCs for Analog and Digital Signal Simulation)
Controller Type: Real PLC (Siemens S7–300).	Controller Type: Real PLC (Allen-Bradley MicroLogix 1400).
Types of Signals Covered: Limited coverage of I/O signals (mainly digital only).	Types of Signals Covered: Full coverage of I/O signals (AI, AO, DI, DO).
Application Level: Educational/Training setups (FESTO FMS, Simulation).	Application Level: Real industrial.
No quantitative metrics reported.	(Noise, Response Time, Average Response Time, Response Accuracy, and Error) are quantitative metrics reported.
Communication Protocol: OPC-UA protocol.	Communication Protocol: Ethernet/IP protocol.
Integration With Node-RED Dashboard visualization only.	Integration With Node-RED Complete dashboard + real- time control + AI–Python scripts.
Cost Efficiency: high (requires OPC-UA servers license in most cases + real PLC).	Cost Efficiency: Medium (open-source tools + real PLC).
Limited scalability.	The integration of Node-RED with PLC offers high scalability.
Novel Contribution: Mainly communication-oriented contribution.	Novel Contribution: Provide a full simulator of analog and digital I/O signals for PLC in industrial systems.
Target Audience: Academic and educational environments.	Target Audience: Industry 4.0 engineers, automation researchers.

## Methods

### Simulation design

**Figure 2.**
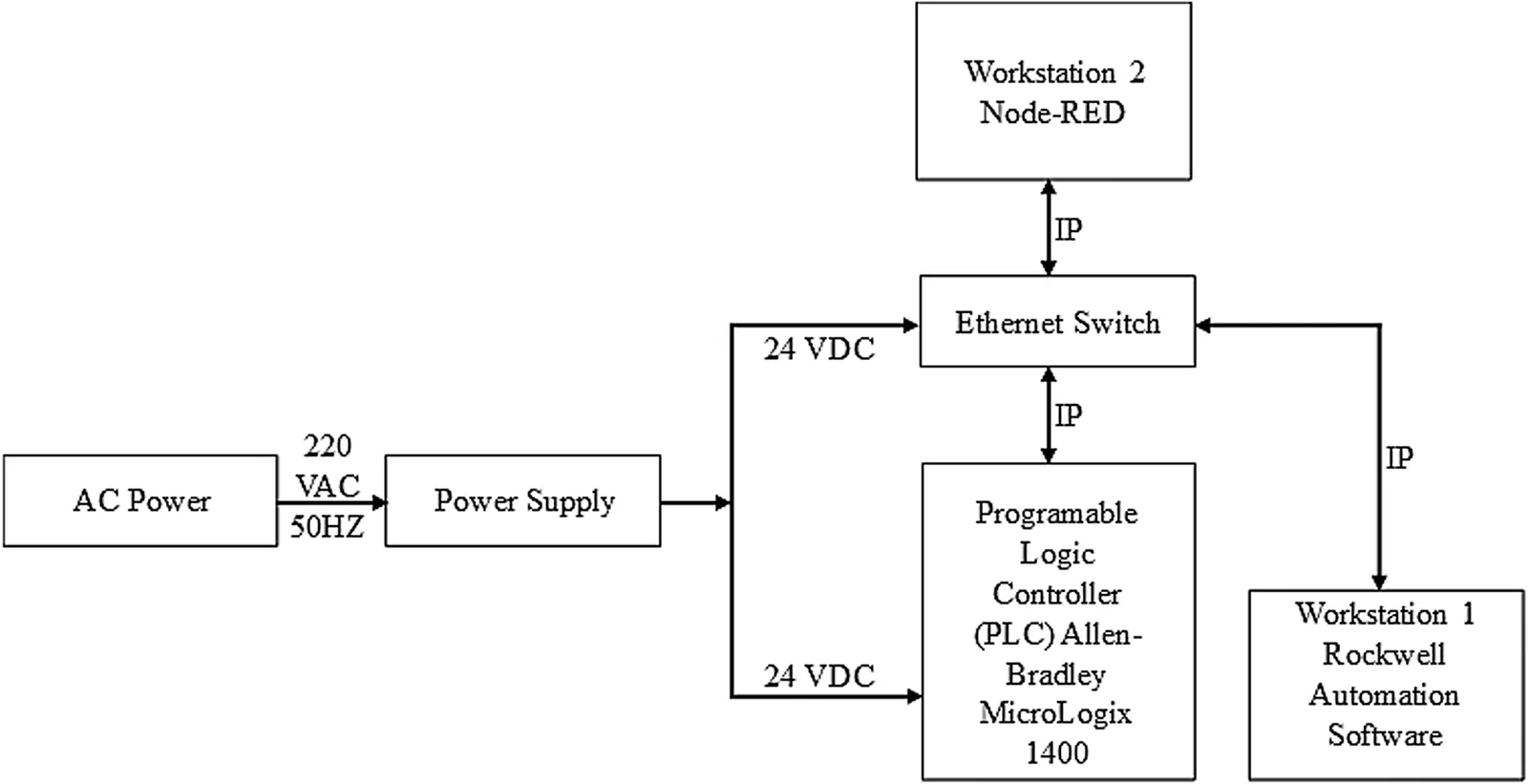
The Block Diagram of the System: This figure illustrates the components of the system, beginning with the AC power source that supplies the DC power supply. This DC power supply, in turn, provides 24 V DC power to both the Ethernet switch and the PLC. Additionally, the figure depicts the network connection between these components via an Internet Protocol (IP) address.

**Figure 3.**
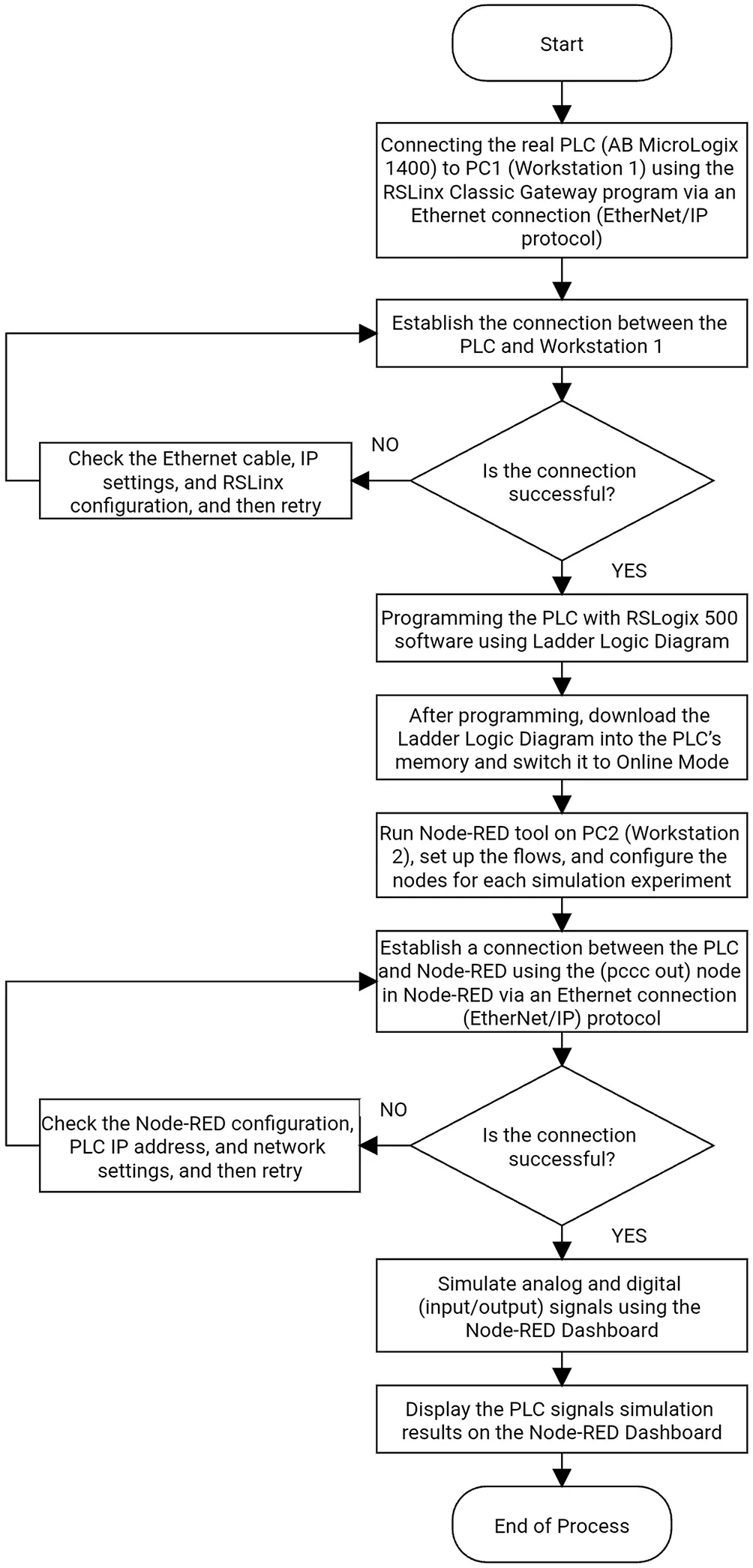
Flowchart of the System: This figure illustrates the steps involved in establishing a connection between a real PLC (Allen-Bradley MicroLogix 1400) and PC1 (Rockwell Automation software). Additionally, it details the establishment of another connection between the programmed PLC and PC2 (Node-RED) to simulate both analog and digital signals, allowing the results to be viewed on the Node-RED dashboard.

**Figure 4.**
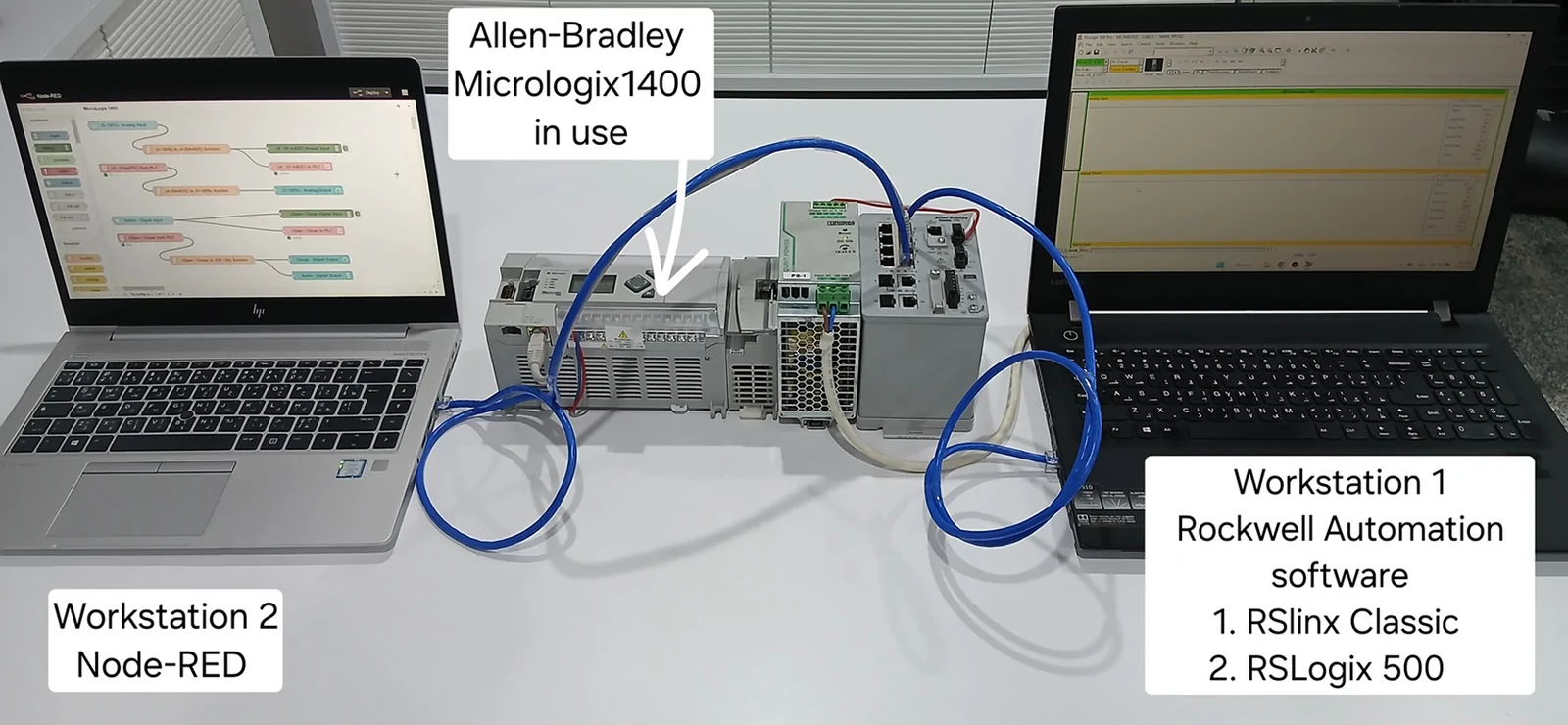
Experimental Setup of Node-RED with MicroLogix 1400 PLC: This figure shows the real system components in the on state, noting the connections between them and the information transmitted and received among them.

The Allen-Bradley MicroLogix 1400 ^21^ PLC was utilized, with specifications in Table 2. The PLC was Ethernet-connected to PC1 (Workstation 1) using RSLinx Classic Gateway (Revision 2.52.00.17 (CPR9)). For PLC programming using Ladder Logic, Workstation 1 included RSLogix 500 (version 9.00.00 (CPR9)) which consists of ladders, rungs, bit, timer/counter, input/output, and other instructions for analog and digital input and output signals. PC2 (Workstation 2) used Node-RED (v4.0.8) to simulate and monitor signals. The integration is achieved via the EtherNet/IP protocol, Node-RED used pccc in/out (Programmable Controller Communication Commands) nodes to connect with the PLC via Python code with other nodes to simulate analog and digital signals. Additionally, the Node-RED Dashboard offered a simple graphical interface for real-time monitoring and management. Further details about the experimental setup are illustrated by the block diagram shown in Figure 2, the flowchart represented in Figure 3, and other attached images included in Figure 4.

**Table 2.** Specifications of allen-bradley microLogix 1400 PLC: This table shows the manufacturing specification for the PLC unit that was used in the simulation experiments for this study.

Model	Power supply	Input	Output
1766-L32BXB	24 V DC	12 Fast 24 V DC, 8 Normal 24 V DC	6 Relay, 3 Fast DC, 3 Normal DC

## Results

**Figure 5.**
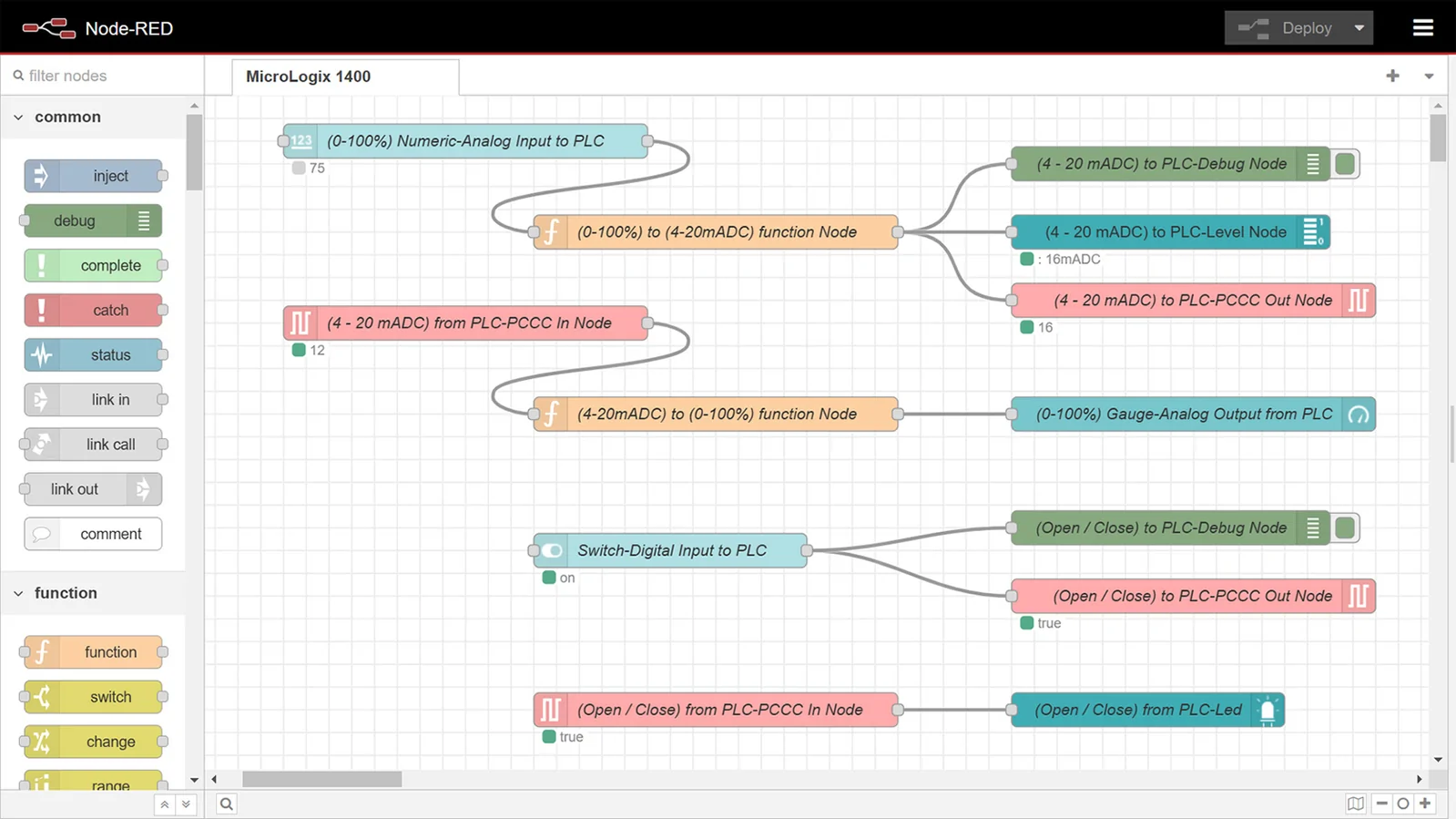
Node-RED Flows for a Real PLC (AB MicroLogix 1400): This figure shows the generated Node-RED flows, where the first flow represents a simulation of the analog input signal, the second flow represents a simulation of the analog output signal, the third flow represents a simulation of the digital input signal, and the fourth flow represents a simulation of the digital output signal.

**Figure 6.**
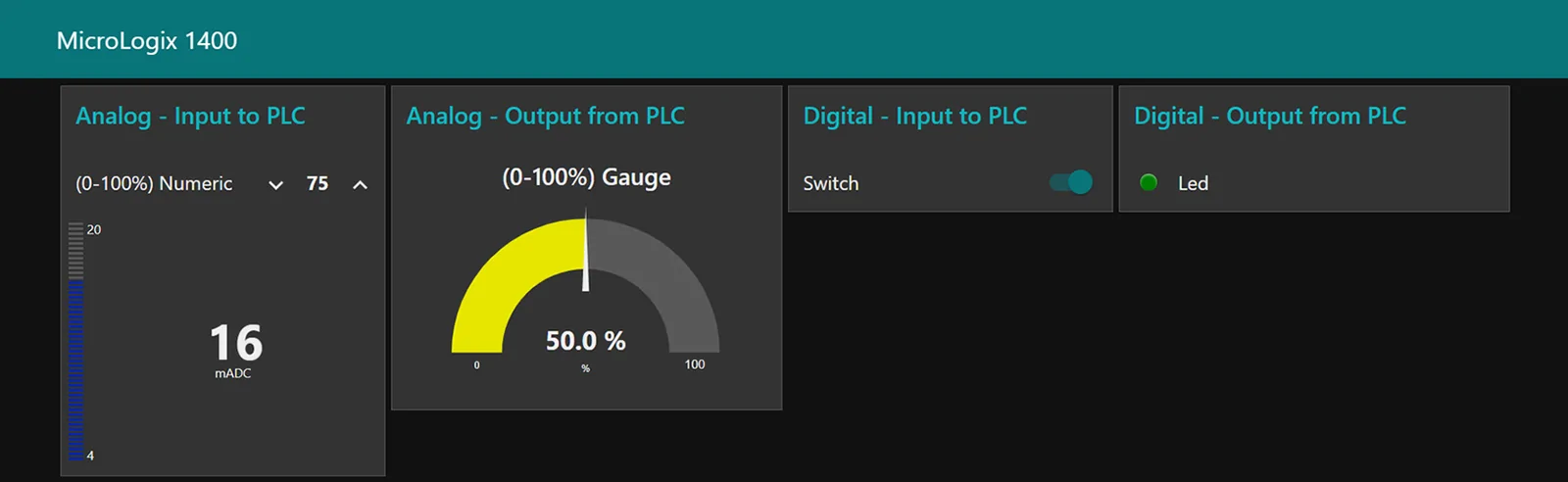
Node-RED Dashboard for a Real PLC (AB MicroLogix 1400): This figure shows the Node-RED dashboard. The first flow simulates the analog input signal to the PLC. It is important to note that the input signal is manually sent to the AB MicroLogix 1400 at 75% of the 0–100% range, which corresponds to 16 mA DC within the 4–20 mA DC range. The second flow simulates the analog output signal, which sets the PLC analog output signal to 50% of the 0–100% range. The third flow simulates the digital input signal; in this flow, the switch is in the ON (true) state. The fourth flow simulates the digital output signal from the PLC, where the LED is illuminated because the PLC’s digital output signal is true.

A. In this research, promising and practical results were achieved when using a real PLC (AB MicroLogix 1400). The simulation was proceeded by the Node-RED platform for the four fundamental PLC signal types as follows:

1. Analog Input Signal Simulation (AI Simulation):
  In this simulation, a numeric node in the Node-RED flow—which is controlled by the Node-RED dashboard—was used as an analog input sensor to manually set the values. Furthermore, the AI signal simulation can function automatically, either randomly or following an increasing-decreasing pattern, by using an inject node in the Node-RED flow. These nodes send an analog input signal between (0–100%). This signal was then converted using a function node in the same Node-RED flow to a DC signal between (4–20) mA DC, which is the standard range compatible with the analog input channel for the PLC. This signal was then transmitted to the real PLC (AB MicroLogix 1400) at the internal analog input channel, such as N7:0, via the (pccc out) node in the same flow. The first flow in Figure 5 illustrates the manual simulation method, and Figure 6 shows the Node-RED dashboard for the four PLC signal types.
2. Analog Output Signal Simulation (AO Simulation):
  In this simulation, an analog output signal was transmitted from the internal analog output channel of the real PLC (AB MicroLogix 1400), such as N7:1—which is controlled by the ladder logic diagram (RSLogix 500)—via the (pccc in) node in the Node-RED flow. This signal, ranging from (4–20) mA DC, was converted using a function node in the same Node-RED flow into a percentage value between (0–100%). This analog value was then sent to the gauge node in the same flow to display the value visually for real-time monitoring of the signal status via the Node-RED dashboard. As shown in the second flow in Figure 5 and Figure 6, which shows the Node-RED dashboard for the four PLC signal types.
3. Digital Input Signal Simulation (DI Simulation):
  In this simulation, the switch node in the Node-Red flow—which is controlled by the Node-RED dashboard—was used to send a digital signal to the real PLC (AB MicroLogix 1400). This digital signal must be either 0 (off/false = open) or 1 (on/true = close) and was sent to the (AB MicroLogix 1400) PLC at the internal digital input channel, such as B3:0/0, via the (pccc out) node in the Node-Red flow. The state of the switch node can be changed manually via the Node-Red dashboard to simulate a real digital input sensor. As shown in the third flow in Figure 5 and Figure 6, which shows the Node-RED dashboard for the four PLC signal types.
4. Digital Output Signal Simulation (DO Simulation):
  In this simulation experiment, a digital signal was received from the internal digital output channel of the real PLC (AB MicroLogix 1400), such as B3:0/3—which is controlled by the ladder logic diagram (RSLogix 500)—via the (pccc in) node in the Node-Red flow. This signal must be either 0 (off/false = open) or 1 (on/true = close). This digital value was then mapped to an LED node in the same flow to display the output signal status visually (either illuminated or off ) via the Node-RED Dashboard. As shown in the fourth flow in Figure 5 and Figure 6, which shows the Node-RED dashboard for the four PLC signal types.

B. The real PLC (AB MicroLogix 1400) was integrated with Industry 4.0 technologies, achieving advanced analysis and enhanced efficiency by merging industrial systems with Industrial Internet of Things (IIoT) technologies represented by the Node-RED environment.
C. Table 3 shows the experimental results (actual PLC reading) that simulate standard values for the analog input signal compared with theoretical values.
D. Table 4 shows the response time was calculated based on simulation experiments.
E. Table 5 shows the response accuracy was calculated based on simulation experiments.

**Table 3.** Comparation between actual values and theoretical values for the analog input signal simulation. From this table, the average deviation between theoretical and actual values approximately (±0.2) mA DC due to the noise issues.

Experiment No.	Standard values of AI signal (%)	Theoretical values (mA DC)	PLC reading values (mA DC)
1	0	4	4.1
2	25	8	7.6
3	50	12	11.8
4	75	16	16.3
5	100	20	20

**Table 4.** The response time and average response time for the four fundamental plc signal types that were simulated. The processing type affects the average response time; digital signals transmit more quickly than analog signals, which need more processing and conversion to/from (4–20) mA DC signals.

Experiment No.	Signal type	Response time (msec)	Average response time (msec)
1	Analog input	80	75–85
2	Analog output	90	85–95
3	Digital input	45	40–50
4	Digital output	35	30–40

**Table 5.** The Response accuracy for the four fundamental plc signal types that were simulated. The response accuracy results indicate that the DI and DO signals exhibit the highest accuracy due to their binary characteristics, while the accuracy of the AI and AO signals is marginally reduced due to signal conversion processes and a (4–20) mA DC fluctuation; however, all results remain within the optimal parameters for industrial applications.

Experiment No.	Signal type	Response accuracy (%)	Error (%)
1	Analog input	98.5	1.5
2	Analog output	98.2	1.8
3	Digital input	99.8	0.2
4	Digital output	99.6	0.4

## Case studies

### Case study 1: AI signal manual simulation method

**Figure 7.**
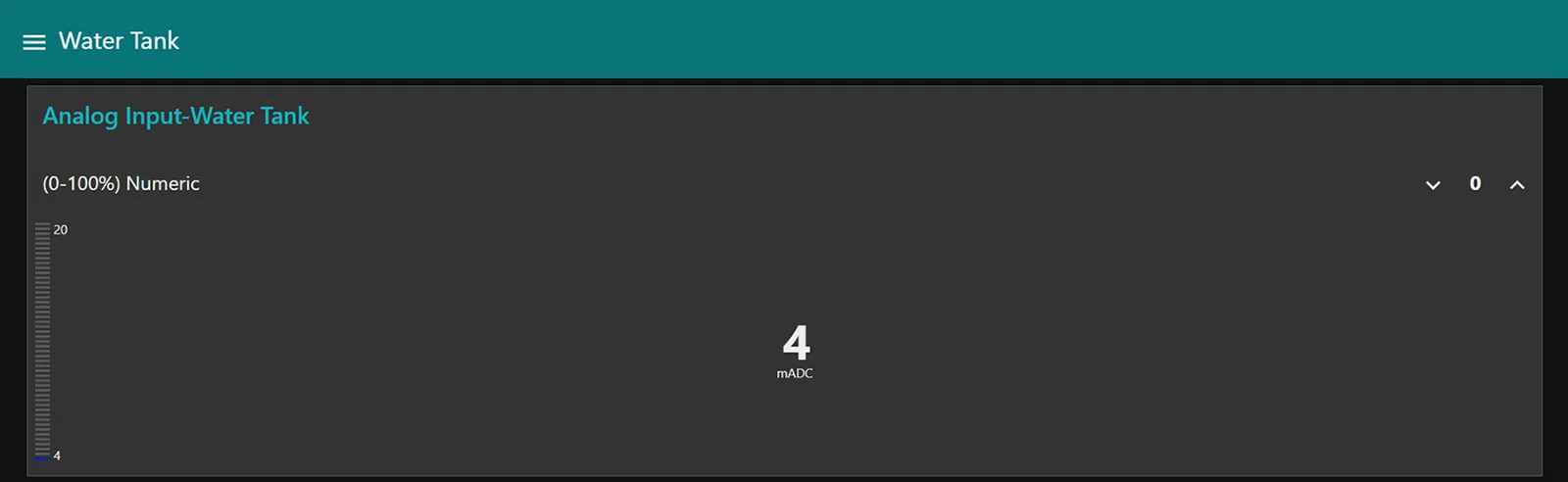
Node-RED Dashboard for an Empty Tank Case: In this figure, a 4 mA DC signal was sent from the Node-RED dashboard as a simulator, which represents 0%, to the PLC, meaning the tank is empty.

**Figure 8.**
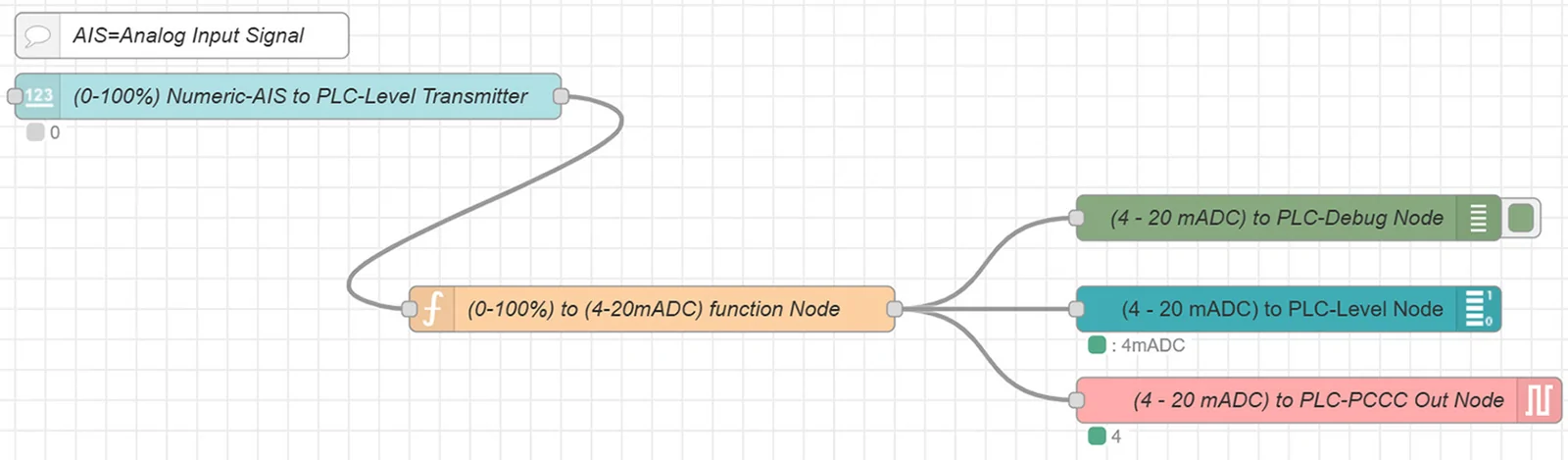
Node-RED Flow for an Empty Tank Case: In this figure, a numeric node was used to manually send a numerical value between 0% and 100% to the real PLC (AB MicroLogix 1400). A function node was then used, and JavaScript code was written to convert the numeric value from 0% to 100% to 4 to 20 mA DC. The signal, since the tank is empty, is 0%, which corresponds to 4 mA DC and was sent to the PLC via the PCCC OUT node. The signal sent to the PLC can be viewed via the Debug node in the Node-RED sidebar, and the signal sent to the PLC can be viewed via the Level node in the Node-RED dashboard.

**Figure 9.**
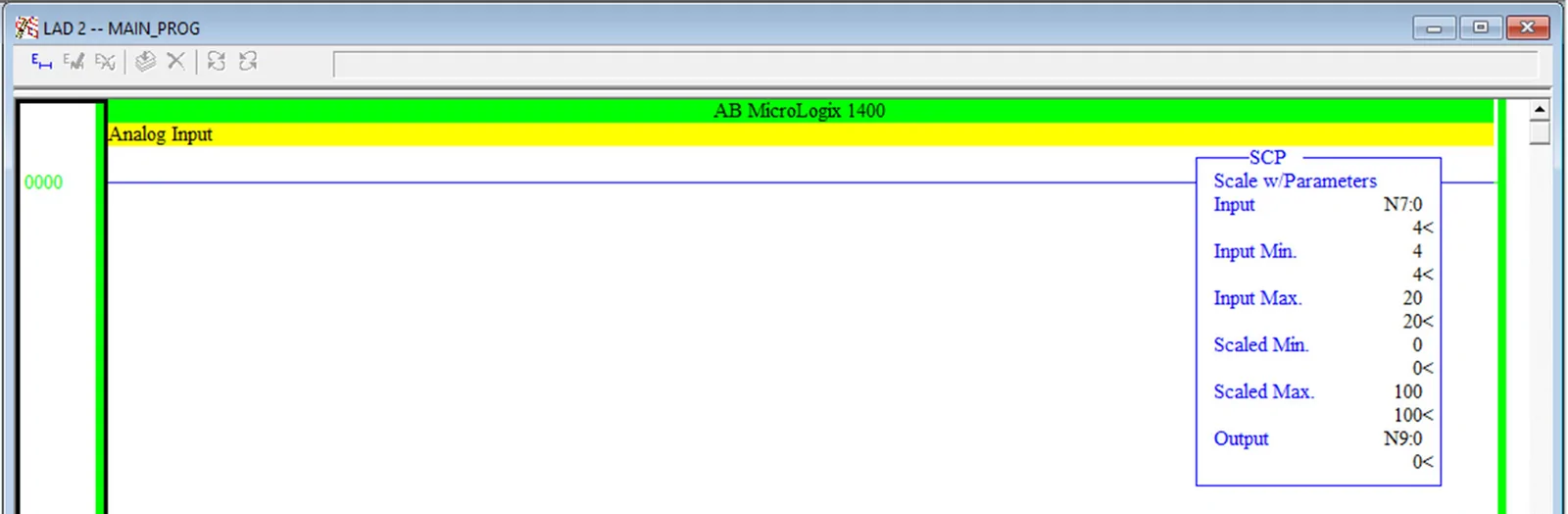
Ladder Logic Program for an Empty Tank Case: This figure represents the RSLogix 500 software interface, which is used to program the Allen Bradley MicroLogix 1400 PLC. Note that the signal received from the Node-RED simulator is 4 mA DC, and it has been converted by the SCP function to 0%, indicating that the tank is completely empty of water.

**Figure 10.**
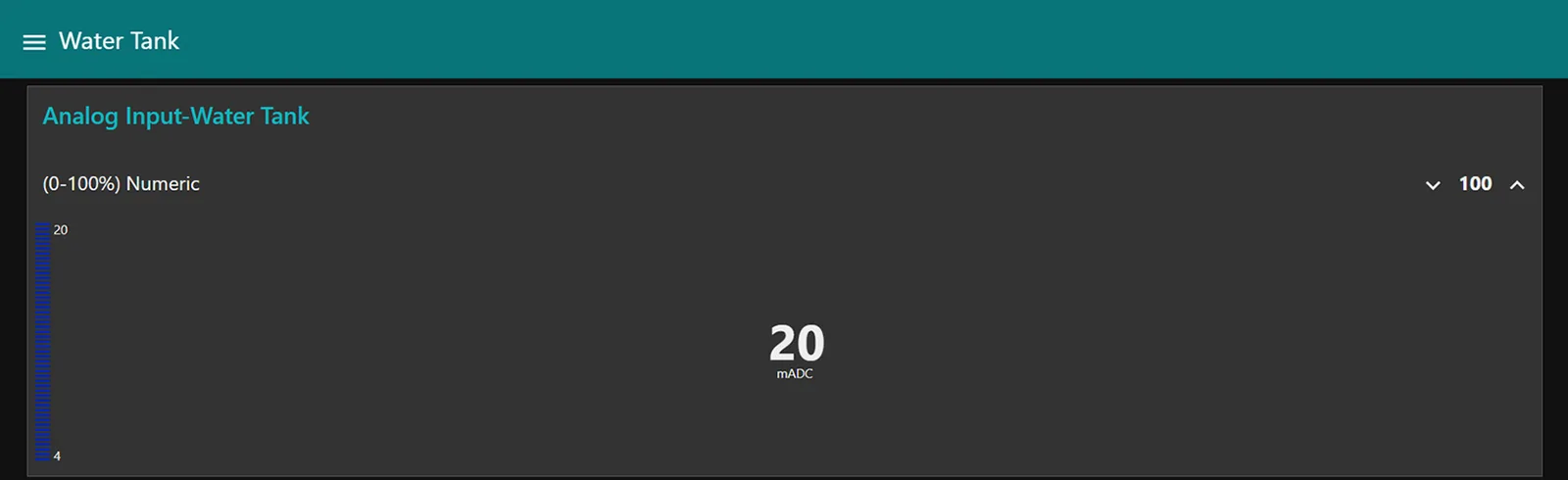
Node-RED Dashboard for a Full Tank Case: In this figure, a 20 mA DC signal was sent from the Node-RED dashboard as a simulator, which represents 100%, to the PLC, meaning the tank is full.

**Figure 11.**
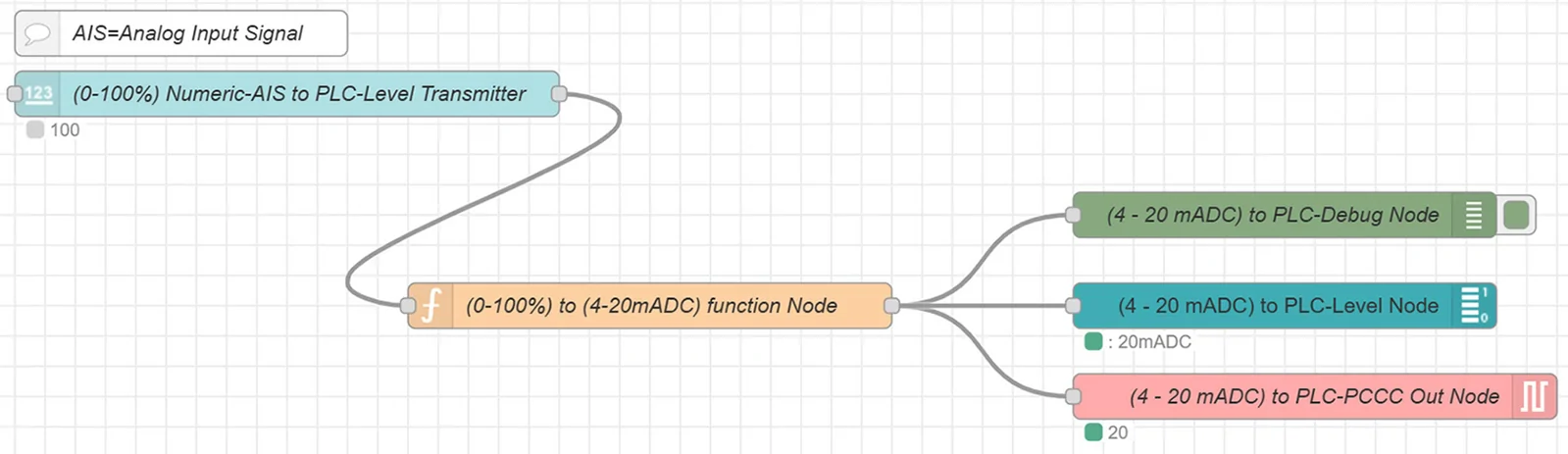
Node-RED Flow for a Full Tank Case: In this figure, a numeric node was used to manually send a numerical value between 0% and 100% to the real PLC (AB MicroLogix 1400). A function node was then used, and JavaScript code was written to convert the numeric value from 0% to 100% to 4 to 20 mA DC. The signal, since the tank is full of water, is 100%, which corresponds to 20 mA DC and was sent to the PLC via the PCCC OUT node. The signal sent to the PLC can be viewed via the Debug node in the Node-RED sidebar, and the signal sent to the PLC can also be viewed via the Level node in the Node-RED dashboard.

**Figure 12.**
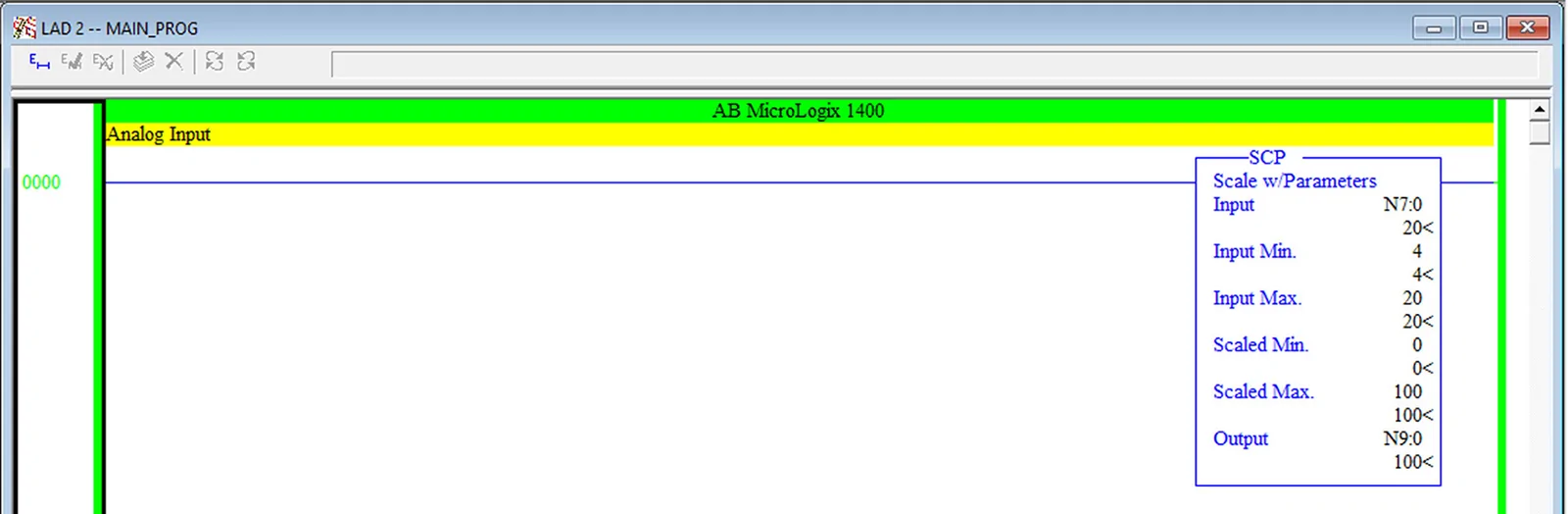
Ladder Logic Program for a Full Tank Case: This figure represents the RSLogix 500 software interface, which is used to program the Allen Bradley MicroLogix 1400 PLC. Note that the signal received from the Node-RED simulator is 20 mA DC, and it has been converted by the SCP function to 100%, indicating that the tank is completely full of water.

**Figure 13.**
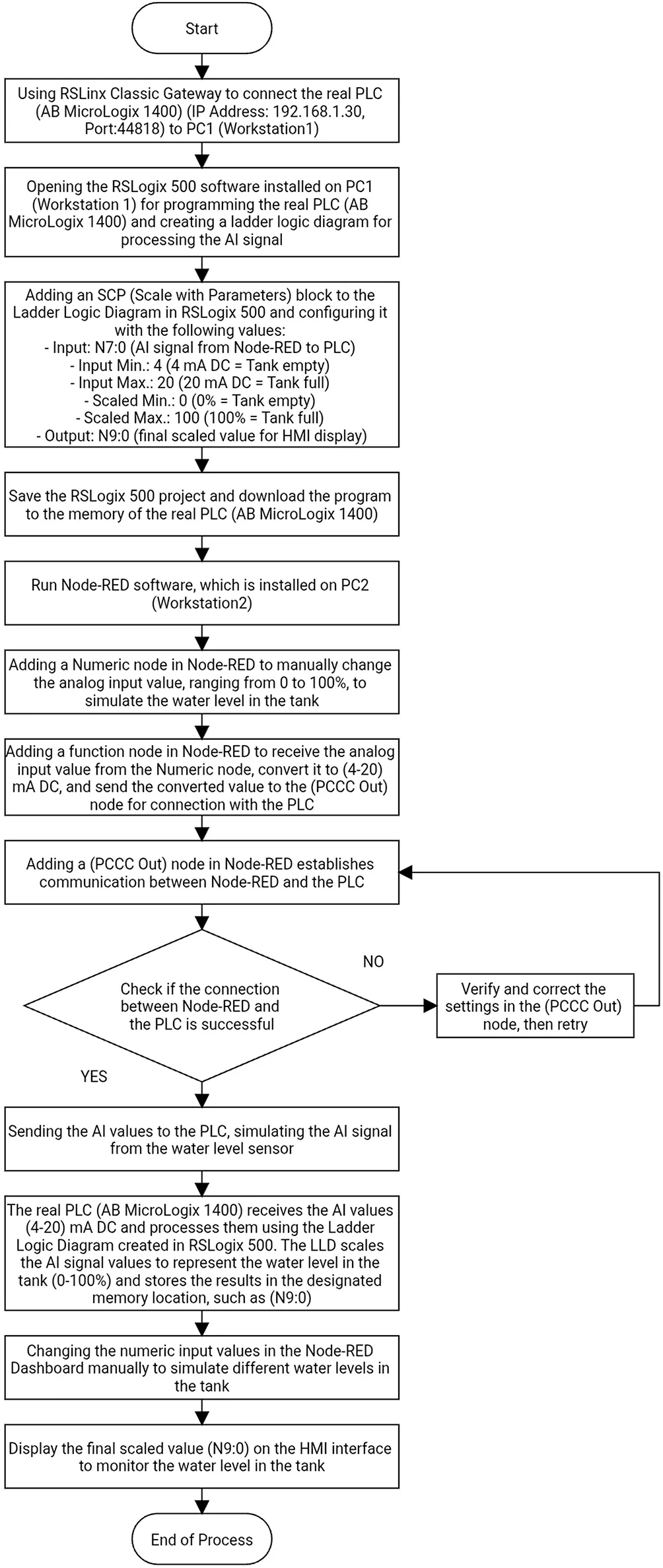
Flowchart for Water Level Case: This figure shows in detail the steps involved in manually simulating an analog input signal, which represents the water level in the tank.

**Figure 14.**
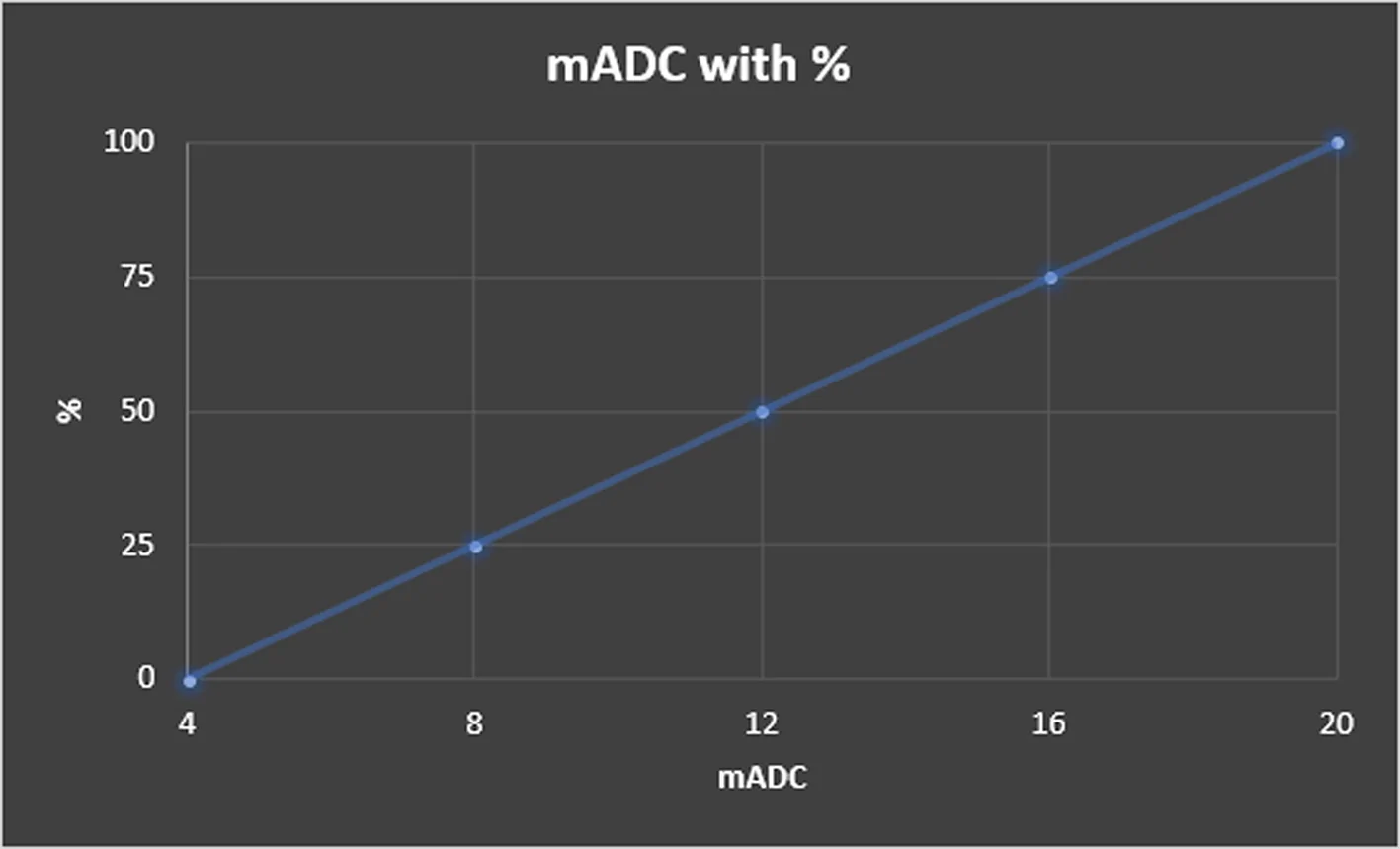
Relationship between the Measured Current Signal (mA DC) and the Water Level Percentage (%) in the tank: It is noted in this figure that the relationship between the signal sent from the Node-RED simulator, whose value ranges from 4 to 20 mA DC, is linear with the water level in the tank, whose value ranges from 0% to 100%.

In the case of an analog input signal representing a water level in the tank, the values of the water level can be simulated manually; the Node-RED environment was used as a standalone simulator. A flow can be constructed to generate a signal representing the water level in the tank, which serves as an analog input signal for a real PLC. This capability is achieved using the numeric node and function node in the Node-RED tool. The numeric node—which is controlled by the Node-RED dashboard—sends an analog input value between (0–100%). This value was then converted using a function node in the same Node-RED flow to (4–20) mA DC, which is the standard range compatible with the analog input channel for the PLC. Finally, this value was transmitted to the real PLC (AB MicroLogix 1400) at the internal analog input channel, such as N7:0, via the (pccc out) node in the same flow. The results can be visualized on the Node-RED dashboard. Figures 7, 8, and 9 illustrate the Node-RED dashboard, Node-RED flow, and ladder logic program, respectively, for the empty tank case. For the full tank case, Figure 10 for the Node-RED dashboard, Figure 11 for the Node-RED flow, and Figure 12 for the ladder logic program. Finally, Figure 13 shows a flowchart for the water level case, and Figure 14 illustrates the relationship between the measured current signal (mA DC) and the percentage of water in the tank.

### Case study 2: AI signal automatic simulation method

**Figure 15.**
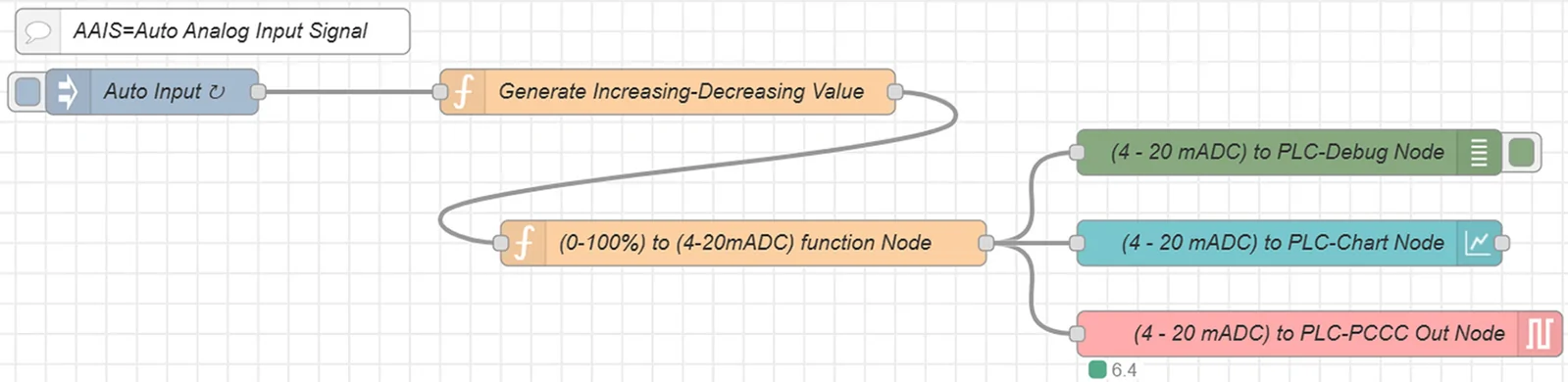
The Flow of Automatic Water Level Simulation: In this figure, an inject node was used to automatically send a numerical value between 0% and 100% to the real PLC (AB MicroLogix 1400). A function node was then used, and JavaScript code was written to simulate the water level in the tank, starting from 0%, gradually increasing to 100%, and then decreasing back from 100% to 0% automatically, and another function node was then used, and JavaScript code was written to convert the numeric value from 0% to 100% to 4 to 20 mA DC. The signal was sent to the PLC via the PCCC OUT node. The signal sent to the PLC can be viewed via the Debug node in the Node-RED sidebar, and the signal sent to the PLC can also be viewed via the Level node in the Node-RED dashboard.

**Figure 16.**
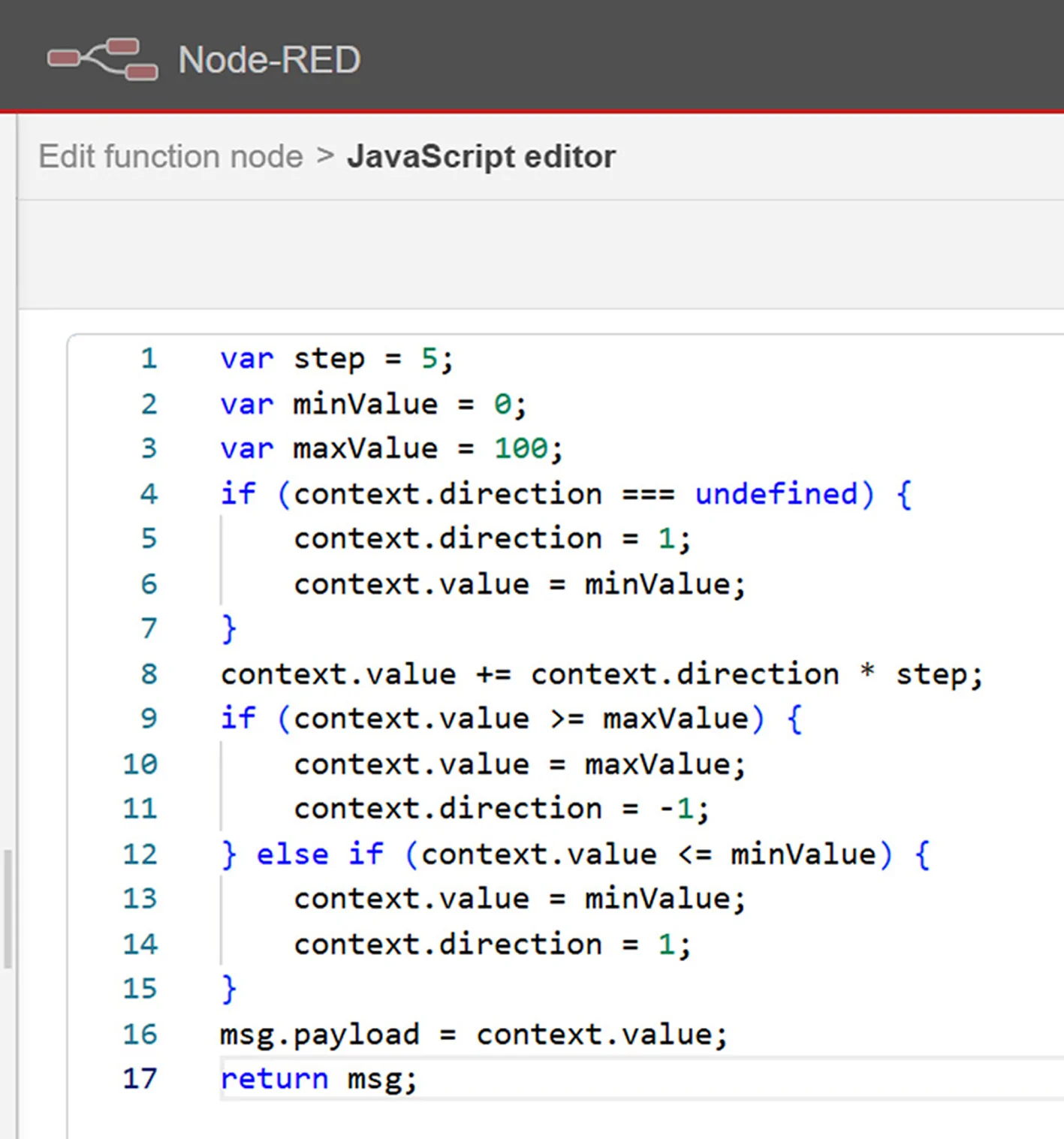
JavaScript Code for Automatic Water Level Simulation: This JavaScript code generates values to simulate the water level in the tank, starting at 0%, gradually increasing to 100%, and then decreasing again from 100% to 0% automatically.

It is also possible to input values that simulate the water level in the tank automatically, either randomly or in an increasing-decreasing pattern, to simulate the real changes in the water level inside the tank. This capability is achieved using the inject node and function node—which is controlled by the Node-RED dashboard—in the Node-RED tool, according to the flow illustrated in Figure 15 and the JavaScript script shown in Figure 16. Through this mechanism, values are generated to simulate the water level in the tank, starting from 0%, gradually increasing to 100%, and then decreasing back from 100% to 0% automatically.

**Figure 17.**
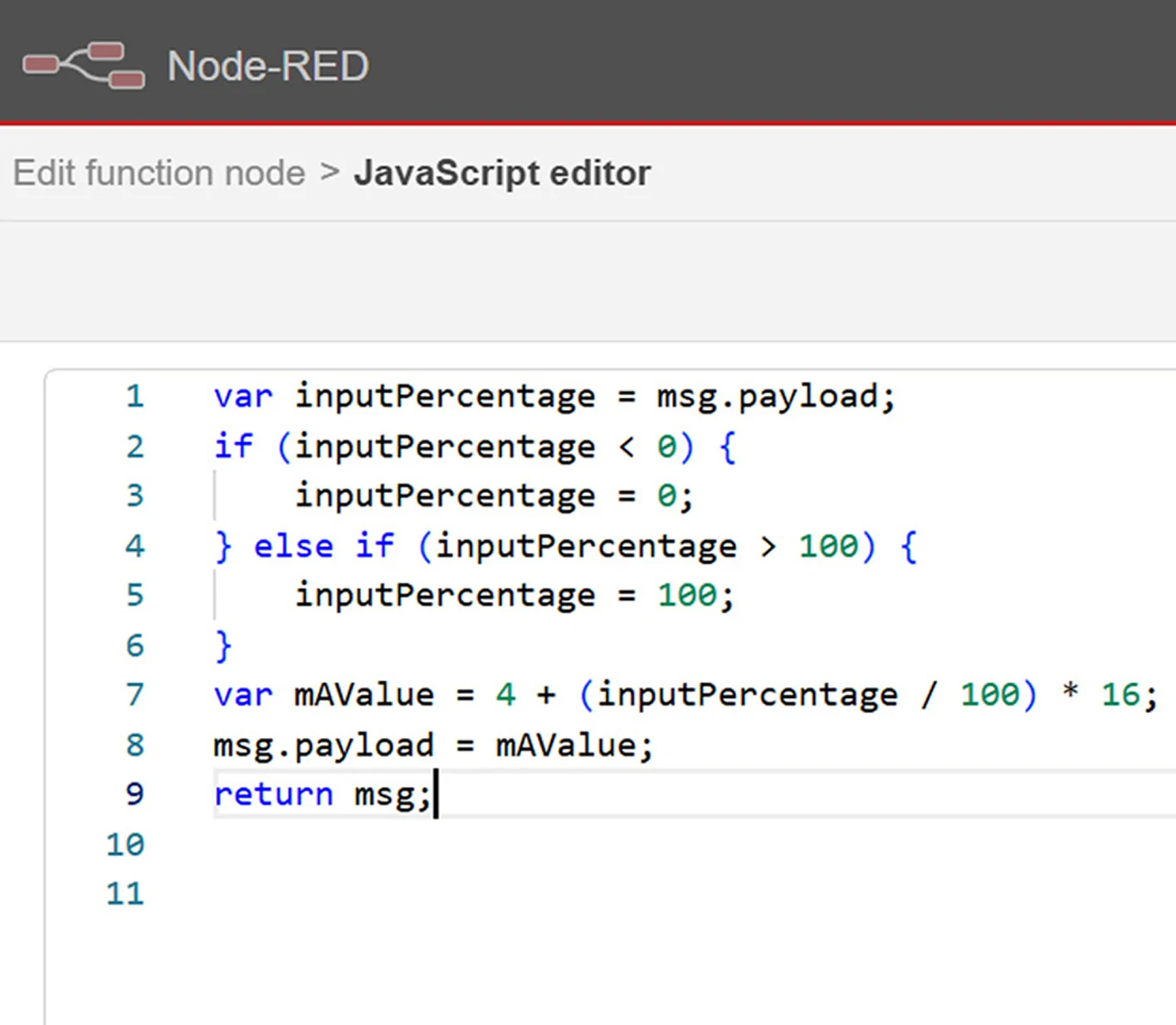
JavaScript Code for Converting Level from % to mA DC: This JavaScript code converts values from percentage (0–100%) to a current signal (4–20 mA DC) and then sends this current signal to the PLC to simulate an analog input signal as if it were sent from a real water level sensor.

**Figure 18.**
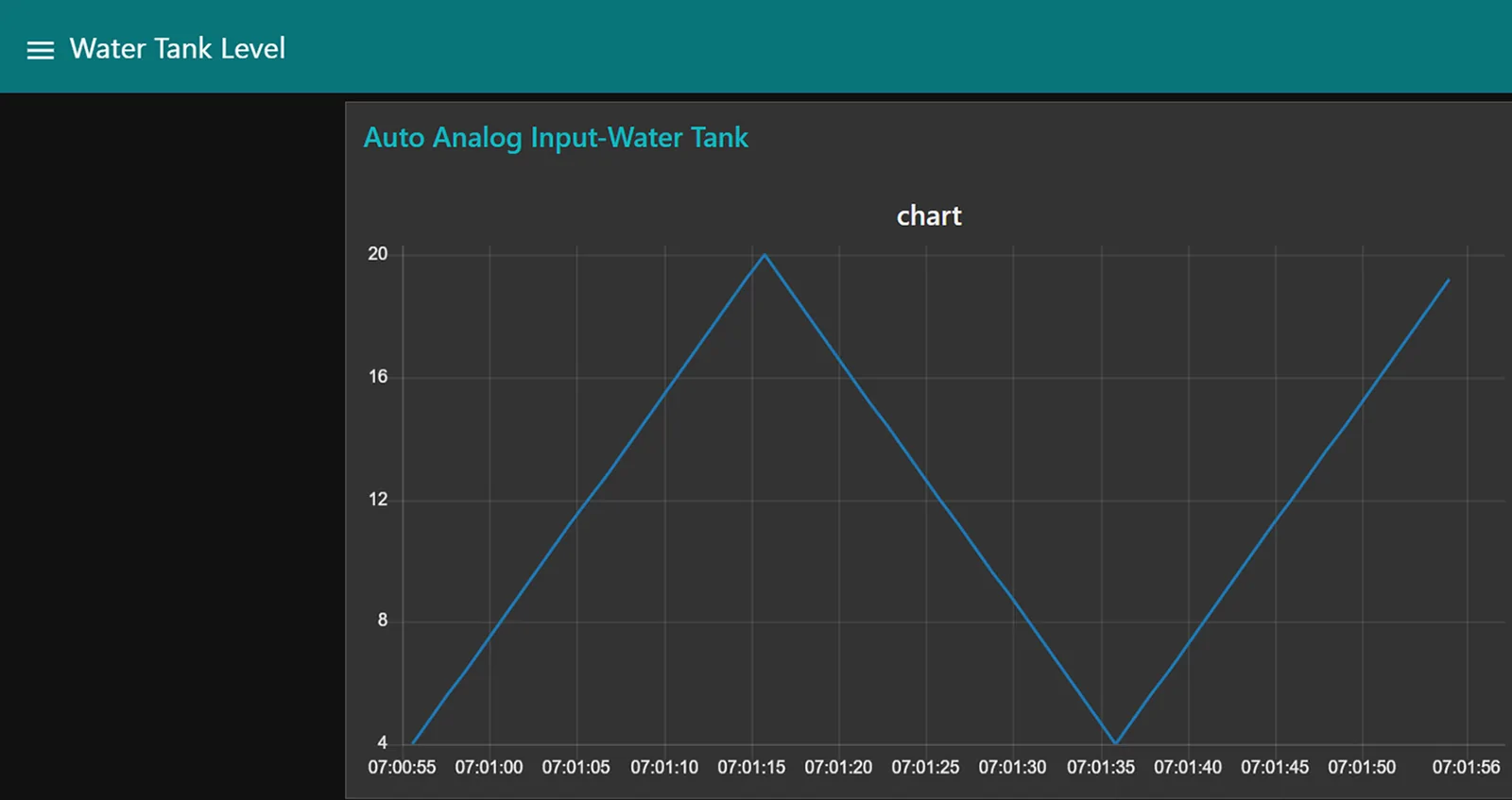
Simulated Current Signal (mA DC) with an Automatic Increasing-Decreasing Pattern: In this chart, the value of the analog input signal can be seen rising from 4 mA DC, meaning the tank is completely empty, to 20 mA DC, meaning the tank is completely filled with water, over time and automatically, repeating the process more than once.

In the same flow, there is another function node, which contains a JavaScript script illustrated in Figure 17, which converts the values from percentage (0–100%) to current signal (4–20 mA DC) and then sends this current signal value to the real PLC (AB MicroLogix 1400) at the internal analog input channel, such as N7:0, via the (pccc out) node in the same flow to simulate the analog input signal produced by a real water level sensor. This allows the system to process the data as if it were coming from an actual sensor, as shown in Figure 18.

### Case study 3: DO signal manual simulation method

**Figure 19.**
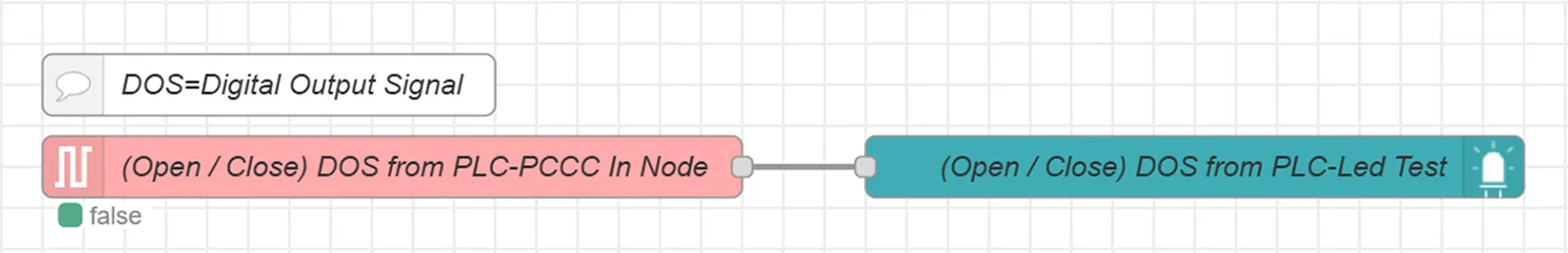
Node-RED Flow for 0 (False/Off ) LED Test Case: In this figure, a digital output signal is sent from the PLC and received by Node-RED via the PCCC In node, with a value of 0, false/off state (the LED is off ). The LED node on the Node-RED dashboard allows you to view the status of the received signal.

**Figure 20.**
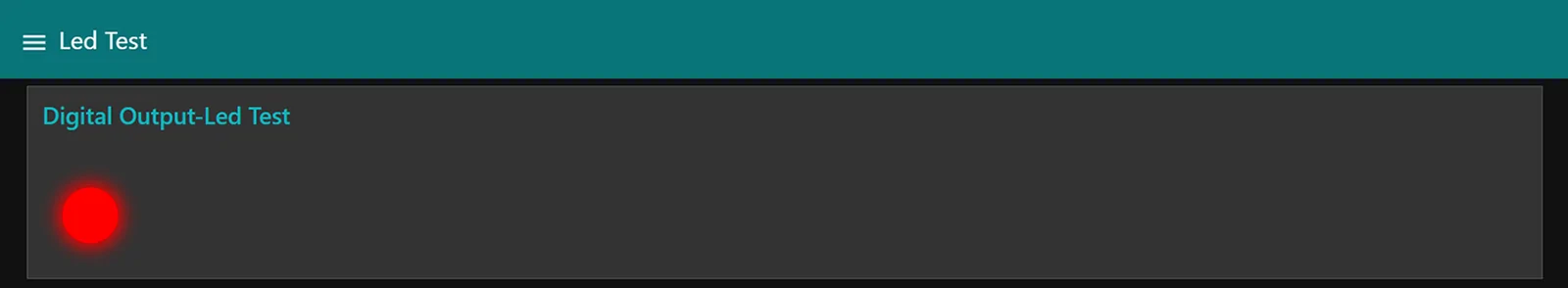
Node-RED Dashboard for 0 (False/Off ) LED Test Case: In this figure, the LED displayed on the Node-RED dashboard is red, which indicates that the LED is off.

**Figure 21.**
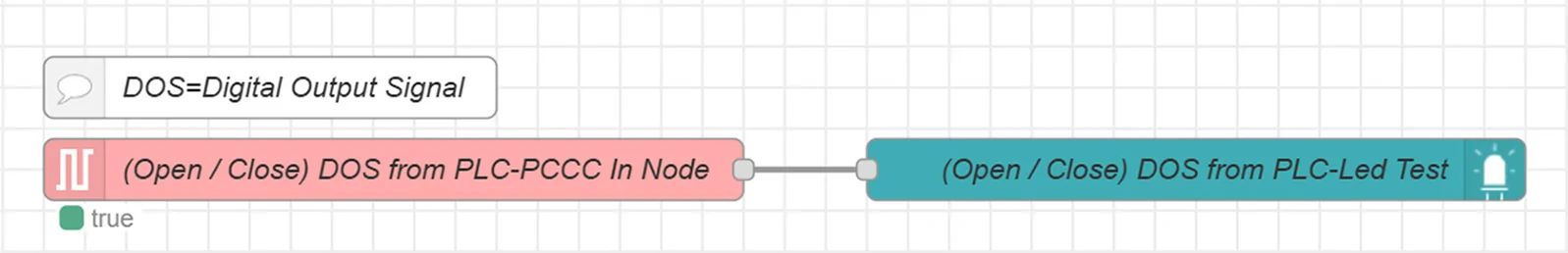
Node-RED Flow for 1 (True/On) LED Test Case: In this figure, a digital output signal is sent from the PLC and received by Node-RED via the PCCC In node, with a value of 1, true/on state (the LED is on). The LED node on the Node-RED dashboard allows you to view the status of the received signal.

**Figure 22.**
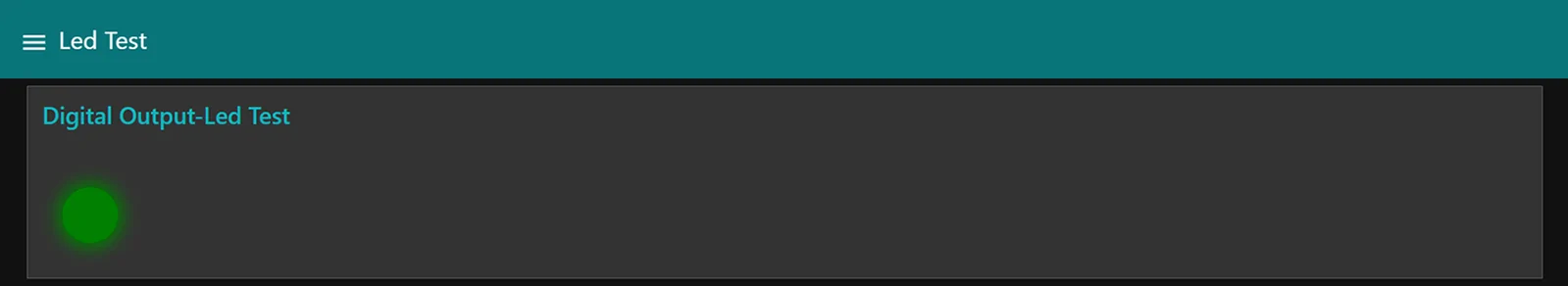
Node-RED Dashboard for 1 (True/On) LED Test Case: In this figure, the LED displayed on the Node-RED dashboard is green, which indicates that the LED is on.

**Figure 23.**
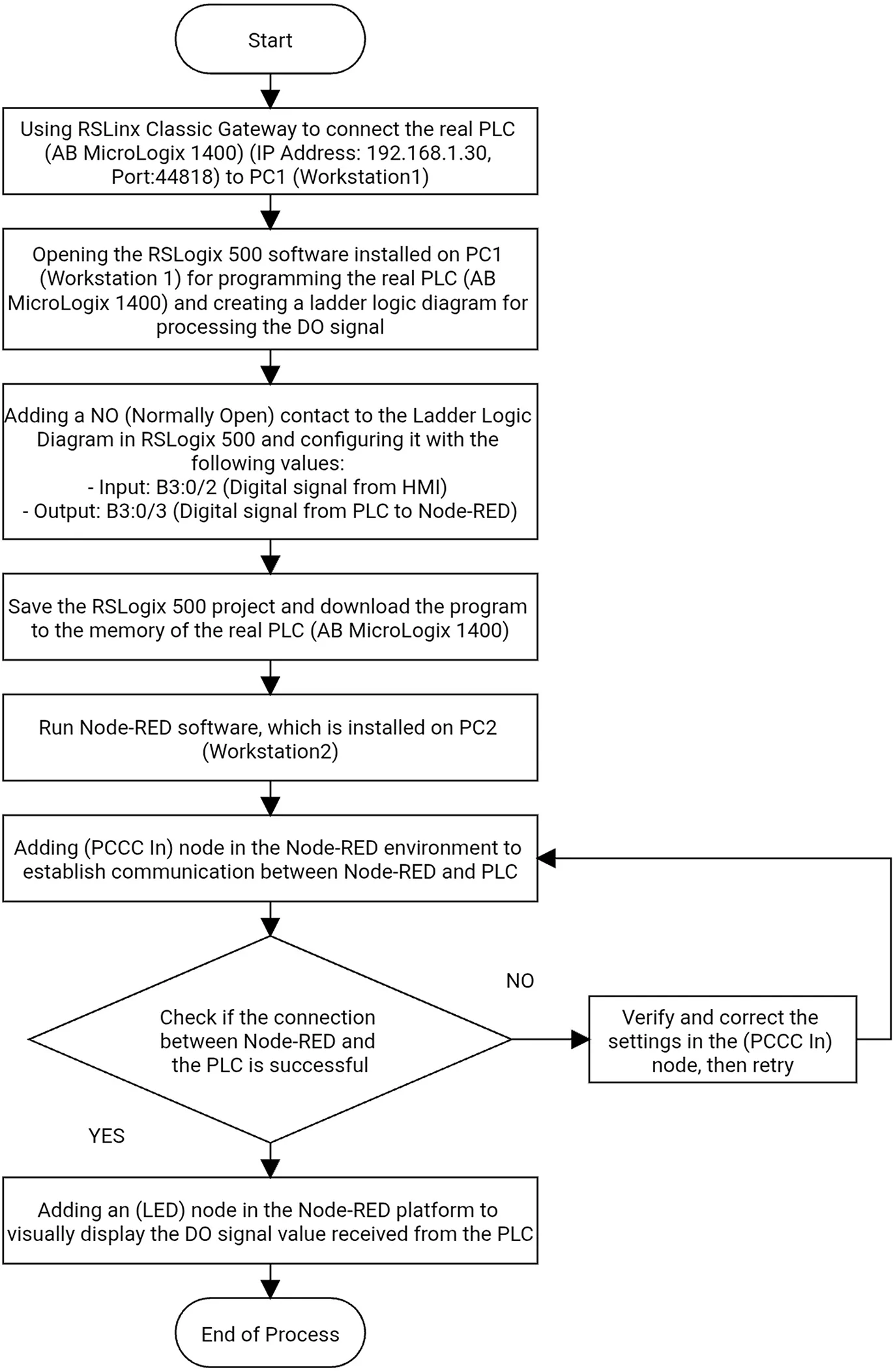
Flowchart for LED Test Case: This figure shows in detail the steps involved in manually simulating a digital output signal, which represents the LED test case.

**Figure 24.**
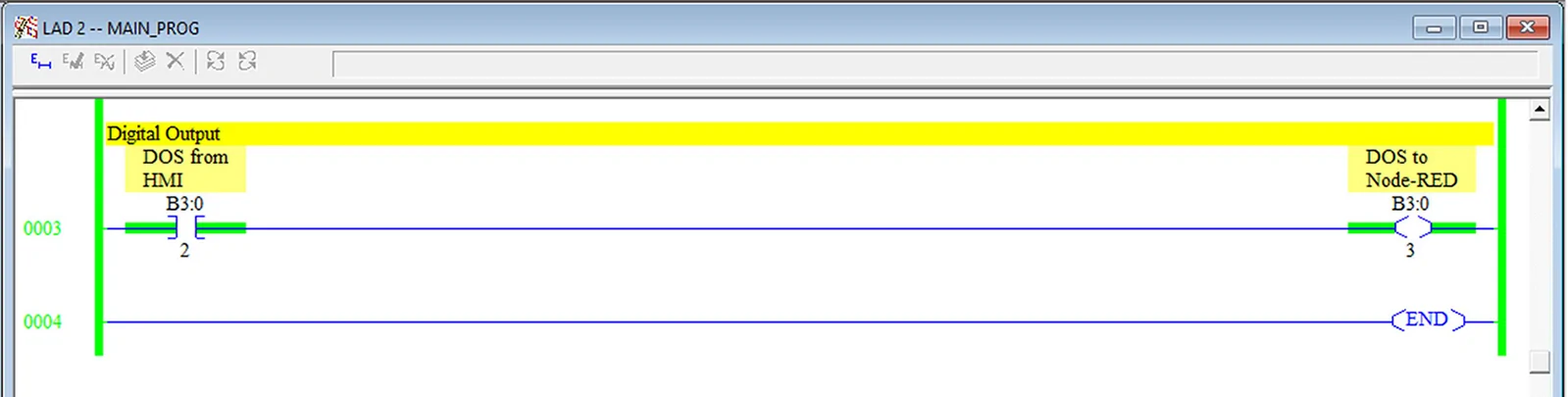
Ladder Logic Program for LED Test Case: This figure represents the RSLogix 500 software interface, which is used to program the Allen Bradley MicroLogix 1400 PLC. Note that the (DOS) digital output signal is sent from the (HMI) human-machine interface to Node-RED with a value of 1 (active), true/on state (the LED is on).

In the case of the LED simulation, a digital signal was received from the internal digital output channel of the real PLC (AB MicroLogix 1400), such as B3:0/3—which is controlled by the ladder logic diagram (RSLogix 500)—via the (pccc in) node in the Node-Red flow. This signal must be either 0 (false/off) or 1 (true/on). This signal was then mapped to the LED node in the Node-RED dashboard, which visually displays the status of the output signal (either on or off). Figures 19 and 20 illustrate the Node-RED flow and the Node-RED dashboard, respectively, for the LED 0 (false/off) test case. For the LED 1 (true/on) test case, Figure 21 for the Node-RED flow and Figure 22 for the Node-RED dashboard. Finally, Figure 23 shows the flowchart of the LED test case, and Figure 24 illustrates the ladder logic program for the LED test case.

## Conclusion

The practical results obtained from simulation experiments, when using a real PLC controller such as the (Allen-Bradley MicroLogix 1400) and connecting it to the Node-RED environment, show that Node-RED offers simulation of analog and digital signals (input/output) either manually or automatically, providing greater flexibility compared to using the traditional PLC simulator (or emulator) alone. Furthermore, you can completely use the Node-RED platform instead of RSLogix Emulate 500, which is the emulator software for Allen-Bradley RSLogix 500, to program controllers and create the basic setup for any automation project. Users can rely entirely on the Node-RED platform. The Node-RED Dashboard simplifies remote monitoring, which facilitates remote automation and decision-making. And in the simulation process, the dashboard successfully sent and received signals to and from the actual PLC (Allen-Bradley MicroLogix 1400) and obtained the required feedback. This is a very important topic, as it allows for testing the automated control system and project controller, identifying strengths and weaknesses, and finding possible solutions before deploying the system in the real world.

### Future work

Future research will focus on evaluating the performance of the proposed Node-RED simulator in large-scale industrial environments involving multiple PLCs and hundreds or thousands of I/O tags. In addition, future studies will investigate CPU and memory utilization, communication latency under heavy workloads, cloud integration, artificial intelligence applications, predictive maintenance, fault diagnosis, and real-time industrial deployment within Industry 4.0 environments.

## Discussion

Node-RED enhances the efficiency and power of industrial systems based on PLCs by providing an accurate emulator for analog and digital signals (input/output) and integrating data with modern technologies in Industry 4.0. The work on configuring and developing the required flows for Node-RED to suit experiment needs was a major challenge, with significant concern about making the Node-RED software easy to use, connect, maintain, and update.

Finally, the models are practically tested and successfully send and receive signals to and from the real PLC (Allen-Bradley MicroLogix 1400) via the Node-RED Dashboard and receive the necessary feedback.

It is recommended to adopt this technology widely in industrial automation to improve performance and flexibility and achieve better results, with a focus on developing programming skills to ensure successful implementation.

### Ethical considerations

This research did not involve human participants, animals, or any sensitive data. Therefore, ethical approval was not required.

## Data Availability

All data generated and analyzed during this study are openly and publicly available in accordance with F1000Research’s Open Data policy. Zenodo: FMM-NodeRED/Integrating-Node-RED-with-Real-PLCs-for-Analog-and-Digital-Signal-Simulation (v1.1.0). https://doi.org/10.5281/zenodo.18229384 This project contains the following underlying data:
• FMM-NodeRED/Integrating-Node-RED-with-Real-PLCs-for-Analog-and-Digital-Signal-Simulation.-v1.1.0.zip (Contains the Node-RED flows files used in the simulation experiments). ^22^ Data are available under the terms of the MIT License. This study does not involve clinical trials, animal experiments, observational studies, or qualitative research. Therefore, none of the CONSORT, ARRIVE, STROBE, COREQ, or SRQR reporting guidelines apply to this work.
