# Supplementary material for: Enhancing Industrial Automation in Industry 4.0: Integrating Node-RED with Real PLCs for Analog and Digital Signal Simulation.  
Source: F1000Res. 2026 Jul 17;15:617. Originally published 2026 Apr 24. [Version 2] doi: 10.12688/f1000research.176034.2 (PMC13560842; doi:10.12688/f1000research.176034.2)
Supplement: Peer review history

# Reviewer report: Reviewer response for version 2

**DOI:** 10.5256/f1000research.205662.r502135

**Published:** 2026-08-12

**Authors:** Erdem Ilten

**Affiliations:** 1 Balikesir University, Balıkesir, Turkey

The main shortcomings in the paper have been addressed.

Is the work clearly and accurately presented and does it cite the current literature?

Partly

If applicable, is the statistical analysis and its interpretation appropriate?

I cannot comment. A qualified statistician is required.

Are all the source data underlying the results available to ensure full reproducibility?

Partly

Is the study design appropriate and is the work technically sound?

Partly

Are the conclusions drawn adequately supported by the results?

Yes

Are sufficient details of methods and analysis provided to allow replication by others?

Yes

Reviewer Expertise:

PLC, automation, embedded systems, electrical machines

I confirm that I have read this submission and believe that I have an appropriate level of expertise to confirm that it is of an acceptable scientific standard.

# Reviewer report: Reviewer response for version 1

**DOI:** 10.5256/f1000research.194062.r479933

**Published:** 2026-05-14

**Authors:** Erdem Ilten

**Affiliations:** 1 Balikesir University, Balıkesir, Turkey

This research paper examines the integration of the Node-RED open-source platform with a real Programmable Logic Controller (PLC) within the framework of Industry 4.0, and its role in analog/digital signal simulation. It demonstrates, with scientific data, that Node-RED can be used as a low-cost, scalable, and reliable test/simulation tool in industrial automation systems.

 Please revise the article according to the suggestions below:

 1- Node-RED and PLC integration is a topic that has been studied before in the literature. Although the article states that it makes a difference by adding "analog signal simulation," the theoretical contribution of this to the academic literature needs to be framed more clearly and strongly.

 2- Although the article discusses the advantages of using Node-RED, it does not offer a technical comparison with other simulation and visualization tools on the market (e.g., Factory IO, Ignition SCADA, or PLC manufacturers' own simulator software). Quantitative data should be used to support the question of in which situations Node-RED is superior or inferior to these tools.

 3- Experiments were conducted using a single PLC (MicroLogix 1400) and a limited number of I/O signals. Analyze the impact of Node-RED on CPU/RAM usage and processing speed in a complex industrial plant scenario where hundreds or thousands of variables (tags) are simulated simultaneously.

 4- Please mention future work in the Conclusion section.

Is the work clearly and accurately presented and does it cite the current literature?

Partly

If applicable, is the statistical analysis and its interpretation appropriate?

I cannot comment. A qualified statistician is required.

Are all the source data underlying the results available to ensure full reproducibility?

Partly

Is the study design appropriate and is the work technically sound?

Partly

Are the conclusions drawn adequately supported by the results?

Yes

Are sufficient details of methods and analysis provided to allow replication by others?

Yes

Reviewer Expertise:

PLC, automation, embedded systems, electrical machines

I confirm that I have read this submission and believe that I have an appropriate level of expertise to confirm that it is of an acceptable scientific standard, however I have significant reservations, as outlined above.

# Reviewer report: Reviewer response for version 1

**DOI:** 10.5256/f1000research.194062.r479928

**Published:** 2026-05-13

**Authors:** Khalaf S Gaeid

**Affiliations:** 1 Tikrit University, Tikrit, Saladin Governorate, Iraq

1. The abstract clearly presents the motivation and practical relevance of integrating Node-RED with Allen-Bradley PLC systems within the context of Industry 4.0. However, the novelty of the proposed approach compared with existing PLC simulation methods should be emphasized more clearly.
2. The methodology section would benefit from additional technical details regarding the communication protocol used between the MicroLogix 1400 and Node-RED, such as Ethernet/IP, Modbus, or OPC-UA.
3. The results section is generally clear, but quantitative performance indicators (e.g., communication latency, response time, accuracy, or reliability percentage) would strengthen the scientific contribution and validation of the proposed system.
4. The conclusion appropriately highlights the cost-effectiveness and practical value of the system; however, mentioning potential industrial applications or scalability for larger automation systems would improve the impact of the study.
5. The abstract language is understandable, but some grammatical refinements are recommended, particularly in the Results section where the sentence structure can be improved for better readability and academic style.
6. It would be beneficial to clarify whether the proposed simulator supports real-time operation and fault-testing scenarios, which are important aspects in Industry 4.0 and cold commissioning environments.

Is the work clearly and accurately presented and does it cite the current literature?

Yes

If applicable, is the statistical analysis and its interpretation appropriate?

Yes

Are all the source data underlying the results available to ensure full reproducibility?

Yes

Is the study design appropriate and is the work technically sound?

Yes

Are the conclusions drawn adequately supported by the results?

Yes

Are sufficient details of methods and analysis provided to allow replication by others?

Yes

Reviewer Expertise:

control system, AI-based control, Machine drives, Fault tolerant control

I confirm that I have read this submission and believe that I have an appropriate level of expertise to confirm that it is of an acceptable scientific standard.
